# Molecular Phylogeny, Laboratory Rearing, and Karyotype of the Bombycid Moth, *Trilocha varians*

**DOI:** 10.1673/031.012.4901

**Published:** 2012-04-10

**Authors:** Takaaki Daimon, Masaya Yago, Yu-Feng Hsu, Tsuguru Fujii, Yumiko Nakajima, Ryuhei Kokusho, Hiroaki Abe, Susumu Katsuma, Toru Shimada

**Affiliations:** ^1^ Department of Agricultural and Environmental Biology, Graduate School of Agricultural and Life Sciences, The University of Tokyo, Yayoi 1-1-1, Bunkyo-ku, Tokyo 113-8657, Japan; ^2^ National Institute of Agrobiological Sciences, 1–2 Ohwashi, Tsukuba, Ibaraki 305-8634, Japan; ^3^ The University Museum, The University of Tokyo, Hongo 7-3-1, Bunkyo-ku, Tokyo 113-0033, Japan; ^4^ Department of Life Science, National Taiwan Normal University, 88, Ting Chou Rd., Sec 4, Taipei 116, Taiwan; ^5^ Gene Research Center, University of the Ryukyu, Chihara 1, Nishihara-cho, Okinawa 903-0213, Japan; ^6^ Department of Biological Production, Faculty of Agriculture, Tokyo University of Agriculture and Technology, Saiwai-cho 3-5-8, Fuchu, Tokyo 183-8509, Japan

**Keywords:** Bombycini, *Bombyx*, *COI* gene, DDC gene, *Ernolatia*, *Ficus*, *Morus*, silkworm

## Abstract

This study describes the molecular phylogeny, laboratory rearing, and karyotype of a bombycid moth, *Trilocha varians* (F. Walker) (Lepidoptera: Bombycidae), which feeds on leaves of *Ficus* spp. (Rosales: Moraceae). The larvae of this species were collected in Taipei city, Taiwan, and the Ryukyu Archipelago (Ishigaki and Okinawa Islands, Japan). Molecular phylogenetic analyses revealed that *T. varians* belongs to the subfamily Bombycinae, thus showing a close relationship to the domesticated silkworm *Bombyx mori* (L.), a lepidopteran model insect. A laboratory method was developed for rearing *T. varians* and the time required for development from the embryo to adult was determined. From oviposition to adult emergence, the developmental zero was 10.47 °C and total effective temperature was 531.2 day—degrees, i.e., approximately 30 days for one generation when reared at 28 °C. The haploid of *T. varians* consisted of n = 26 chromosomes. In highly polyploid somatic nuclei, females showed a large heterochromatin body, indicating that the sex chromosome system in *T. varians* is WZ/ZZ (female/male). The results of the present study should facilitate the utilization of *T. varians* as a reference species for *B. mori*, thereby leading to a greater understanding of the ecology and evolution of bombycid moths.

## Introduction

The domesticated silkworm, *Bombyx mori* (L.) (Lepidoptera: Bombycidae), is a lepidopteran model insect (Goldsmith et al. 2005). To date, more than 1000 geographic and mutant silkworm strains have been collected and maintained (Tazima 1978; Goldsmith et al. 2005; Banno et al. 2005). Recently, whole genome sequences of *B. mori* and the wild species from which it was derived, *B. mandarina*, were determined (The International Silkworm Genome Consortium 2008; Xia et al. 2009), and a highly efficient system for germline transformation was developed (Tamura et al. 2000). *Bombyx mori* has been used in pioneering studies in insect genetics, physiology, and biochemistry, and there has been research done on almost all of its traits (Tazima 1978; Goldsmith et al. 2005). In contrast, our knowledge of the biology of species related to *B. mori* and *B. mandarina* is very limited.

According to Lemaire and Minet (1998), the family Bombycidae is comprised of four subfamilies: Apatelodinae, Phiditinae, Prismostictinae, and Bombycinae. However, this taxonomy is not supported by recent molecular phylogenetic analyses (Regier et al. 2008a, 2008b; Zwick et al. 2011). The genus *Bombyx* is a member of the subfamily Bombycinae, which includes 20 other genera such as *Gunda, Ocinara, Trilocha, Triuncina, Penicillifera, Bivincula, Ernolatia, Gnanthocinara, Rondotia, Sesquiluna, Colla, Epia*, and *Quentalia* (Lamaire and Minet 1998; Zolotuhin and Witt 2009). Despite these observations, little is known about the biology of species of Bombycinae other than *B. mori* and *B. mandarina*. The aim of the present study was to increase our knowledge of the biology of species related to *B. mori* and to establish a new model species within this group. Given the availability of whole genome data and vast information from previous studies on *B. mori* (Goldsmith et al. 2005; The International Silkworm Genome Consortium 2008), a new model species of Bombicinae (or Bombycini) would serve as a useful reference for *B. mori* and help shed light on the molecular mechanisms underlying ecological traits of bombycid moths, such as host plant selection, tolerance to plant secondary metabolites, resistance to insecticides and pathogens, sex pheromone production and recognition, body and wing color patterns, and diapause systems.

The purpose of this study was to provide basic information on the biology of the bombycid moth *Trilocha varians* (Walker) and facilitate future comparative genomics studies between *T varians* and *B. mori*. Here, the molecular phylogeny, laboratory rearing, and karyotype of *T. varians* are reported. This species is widely distributed in South and Southeast Asia, from India and Nepal through Vietnam, Thailand, Myanmar, southern China, Sumatra, Java, and Taiwan (Zolotuhin and Witt 2009). Recently, adults of this species were recorded for the first time in Japan in the Ryukyu Islands, Japan (Kishida 2002). The larvae feed on leaves of plants belonging to the genus *Ficus* (L.) (Rosales: Moraceae) (Zolotuhin and Witt 2009), and are known as important pests of ornamental and roadside *Ficus* trees, such as *F. benjamina* (Weeping Fig) and *F. microcarpa* (Chinese Banyan). Larvae and pupae of *T. varians* were collected in Taipei city (Taiwan), Ishigaki island (Japan), and the main island of Okinawa (Japan) for the present study. Using these samples, the phylogenetic position of *T. varians* was examined based on nuclear and mitochondrial genes, confirming its close phylogenetic relationship to *B. mori*. Moreover, a laboratory rearing system was established for *T. varians* and calculations of the developmental zero and effective heat units for its developmental period at different temperatures were made. The karyotype and sex chromosome system of *T. varians* were also investigated.

## Materials and Methods

### Collecting and rearing of *T. varians*

Larvae of *T. varians* were collected from *F. microcarpa* trees in Taipei city (25.01° N, 121.54° E), Taiwan, in October 2009; Ishigaki Island (24.36° N, 124.13° E), Japan, in March 2010; and Okinawa Island (26.66° N, 128.10° E), Japan, in November 2010. In Taipei, *T. varians* larvae were easily found on the leaves of roadside *Ficus* trees (approximately 30 larvae could be collected within one hour), while much more effort was required to find them on Ishigaki and Okinawa Islands; only two and 15 larvae were found during a four— day survey on Ishigaki Island and a six—day survey on Okinawa Island, respectively). A laboratory colony from Ishigaki Island was established and maintained in containment facilities in the University of Tokyo, Japan. Larvae were reared on agrochemical—free *F. microcarpa* or *F. superba* leaves under a 12:12 L:D photoperiod. Rearing experiments were performed at a constant 25 °C unless otherwise indicated. Eggs and first instar larvae were reared in sterile plastic Petri dishes (90 × 15 mm) that were tightly sealed with Parafilm. Individuals at other stages were reared in disposable plastic cups (430 mL volume; 1100 mm diameter × 600 mm height). Small ventilation holes were made on the wall of the 430 mL plastic cups with an insect pin.

### DNA extraction, polymerase chain reaction (PCR), and DNA sequencing

The nucleotide sequences of mitochondrial *cytochrome c oxidase I* (*COI*) and nuclear protein—coding gene *dopa decarboxylase* (*DDC*) were selected in our molecular study. The mitochondrial gene is known as a useful DNA marker for analyzing inter— or intra— specific relationships of silkmoths and other lepidopterans (Hebert et al. 2004; Arunkumar et al. 2006), and the nuclear gene has been used in molecular phylogenetic studies of silkmoths at higher systematic levels (Regier et al. 2008a; Zwick et al. 2011).

Mitochondrial DNA was extracted from adult legs and pupal abdomens using the DNeasy Blood and Tissue kit (Qiagen, www.qiagen.com). A fragment of the *COI* gene of *T. varians* (1461 bp) was amplified by polymerase chain reaction (PCR) with primer sets 5′-CGAAAATGAATTTATTCTACAAA TCATA-3′ and 5′- GGTAGTTCATTATATGAATGTTCTGCT G-3′, designed based on the sequence of *B. mori COI* (accession number, AB083339) under the following conditions: 94 °C for one min followed by 32 cycles of 94 °C for 30 sec, 45 °C for 30 sec, and 72 °C for one min. A partial fragment of *COI* gene (597 bp) of *Ernolatia moorei*, collected in Wulai, Taiwan, was also amplified by PCR with primer sets CI-J-1632 (5′-TGATCAAATTTAT AAT-3′) and CI-N-2192 (5′-GGTAAAATTAAAATATAAACTTC-3′) (Kambhampati and Smith 1995) under the same conditions as those described above. The resulting PCR products were purified from gels and directly sequenced using the ABI PRISM Big Dye Terminator Cycle Sequencing Ready Reaction kit ver. 3.0 and analyzed using an ABI3130x1 genetic analyzer (Applied Biosystems, www.appliedbiosystems.com). To avoid inaccurate identification of species, the *COI* sequences obtained were checked using BOLD v2.5 (Barcode of Life Date Systems; http://www.boldsystems.org/views/login.php) (Ratnasingham and Hebert 2007), where DNA barcodes from broad taxa are available.

Total RNAs of *T. varians* were extracted from the entire bodies of second instar larvae at the molting stage using TRIzol reagent (Invitrogen, www.invitrogen.com) as described previously (Daimon et al. 2003; Daimon et al. 2010). Total RNA was reverse— transcribed, diluted, and used for PCR. A first—strand cDNA reaction was performed using the TAKARA RNA PCR kit (AMV) ver. 3.0 (TAKARA BIO Inc., www.takarabio.com) with an oligo dT adaptor primer. PCR was performed under the following conditions: 94 °C for one min followed by 35 cycles of 94 °C for 30 sec, 45 °C for 30 sec, and 72 °C for two min. A partial fragment (1282 bp) of the *DDC* gene was amplified by PCR with a degenerate primer described in previous studies, 1.2F (5′-GARAAYATYAGAGAYAGRCARGT-3′) and 7.5sF (5′-TCCCANGANACRTGVATRTC-3′) (Regier et al. 2008a; Regier et al. 2008b). The PCR products were subcloned into the pGEM-Teasy vector (Promega Corporation, www.promega.com) and sequenced as described previously (Daimon et al. 2003). The DNA and RNA samples used in this study are preserved in the Department of Agricultural and Environmental Biology, Graduate School of Agricultural and Life Sciences, University of Tokyo.

### Phylogenetic analyses

**Figure 1.**
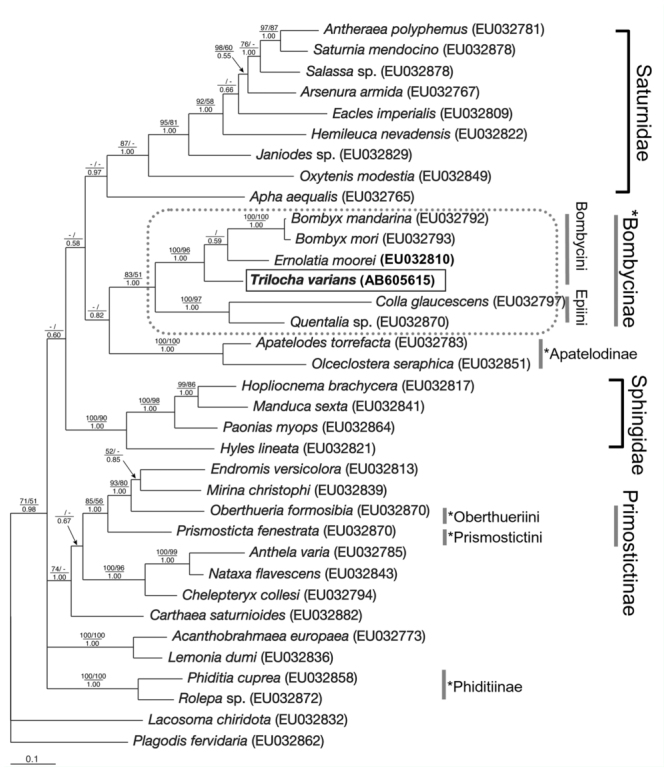
Phylogenetic analysis of Bombycoidea using nuclear *DDC* gene sequences. The topology shows Bayesian inference tree using program MrBayes 3.1.2. *Lacosoma chiridota* and *Plagodis fervidaria* were designated as out-group taxa. Numbers indicate bootstrap values from NJ (top left) and MP (top right) analyses, and posterior probabilities from Bayesian analysis (bottom). Only bootstrap values > 50% and Bayesian posterior probabilities > 0.5 are shown. Branch lengths represent nucleotide substitutions per site. *Trilocha varians* is shown as a box, and the subfamily Bombycinae is shown as a grey dashed box. Accession numbers are indicated in parentheses. Asterisks indicate subgroups of Bombycidae as defined by Minet (1994). High quality figures are available online.

**Figure 2.**
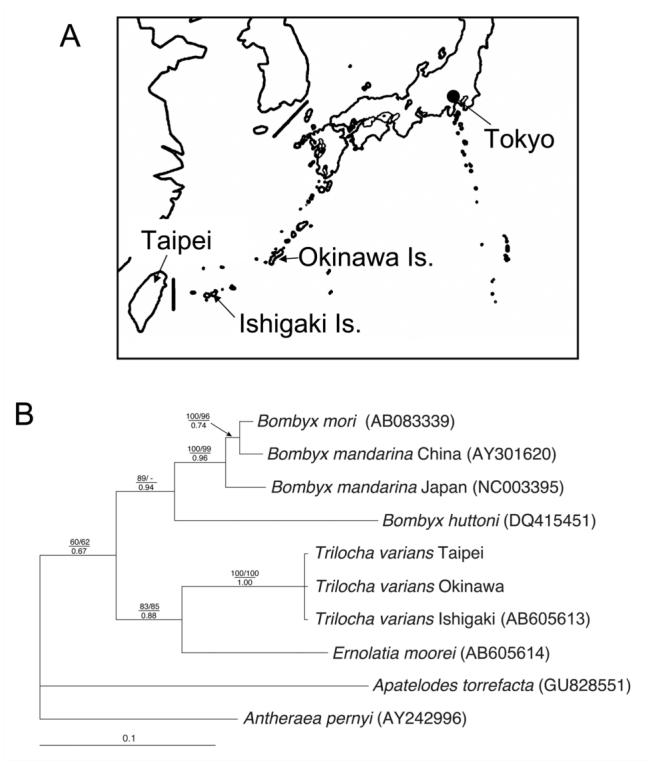
Geographical map showing sampling localities and phylogenetic analysis of Bombycini using mitochondrial *COI* gene sequences. (A) Larvae of *Trilocha varians* were collected in Taipei, Ishigaki Island, and Okinawa Island. (B) The topology shows Bayesian inference tree using program MrBayes 3.1.2. *Apatelodes torrefacta* and *Antheraea pernyi* were used as out-group taxa. Numbers indicate bootstrap values from NJ (top left) and MP (top right) analyses, and posterior probabilities from Bayesian analysis (bottom). Only bootstrap values > 50% and Bayesian posterior probabilities > 0.5 are shown. Branch lengths represent nucleotide substitutions per site. The sequences of *Trilocha varians* were 100% identical among samples from Taipei, Ishigaki Island, and Okinawa Island. Localities and accession numbers are indicated in parentheses. *Bombyx huttoni* (DQ415451) appeared to be erroneously registered by Arunkumar et al. (2006) as *Theophila religiosa*(e)^*^ in GenBank. ^*^ The history of *Theophila religiosae* (Helfer, 1837) is complicated. Moore (1858) and Hutton (1864) treated *Bombyx huttoni* Westwood, 1847 as a junior synonym of *Theophila religiosae*. However, Dierl (1979) inaccurately designated a specimen of the current *Triuncina religiosae* as the neotype of this specific epithet, *religiosae*, as the type specimen of *Theophila religiosae* was lost. Thus, the scientific name of *Bombyx huttoni* was revived instead of *Theophila religiosae*. High quality figures are available online.

In this molecular study, the phylogenetic position of *T. varians* was investigated at two hierarchical levels: (1) within the Bombycoidea (*s. str*.) to examine phylogenetic relationships of *Trilocha*, and (2) within the Bombycinae (Bombycini) to determine the phylogenetic position of *T. varians* and to investigate the genetic diversity of this species. For *DDC* analyses, in addition to the above—mentioned samples, nucleotide sequences were selected of bombycoid taxa and their affinities, which were analyzed in the previous higher—level analysis of Bombycoidea (Regier et al. 2008a). For *COI* analyses, sequence data of all bombycine species and two suitable out—group taxa were used (each species of Saturniidae and Sphingidae) that were registered in GenBank. These species and their GenBank accession numbers are shown in Figures 1 and 2B. The nucleotide sequences of *DDC* and *COI* were aligned using the Clustal × program (Thompson et al. 1997) and the Se-Al Sequence Alignment Program v1.d1. software (Rambaut 1996). Although neither deletions nor insertions were found in the sequence alignment of *COI* gene (520 bp), gaps in the alignment of the *DDC* gene were removed, and the remaining *DDC* sequences (1026 bp) were used for further analyses.

Phylogenetic trees were constructed using neighbor joining (NJ) and maximum parsimony (MP) methods in the program PAUP^*^ 4.0b10 (Swofford 2002), and Bayesian analysis using MrBayes 3.1.2 (Ronquist and Huelsenbeck 2003). The methods used for the phylogenetic analyses mainly followed Yago et al. (2008). For the NJ method, the Kimura two—parameter model was chosen for the first (*DDC*) and second (*COI*) datasets. The MP method was performed using a heuristic search with the TBR swapping algorithm of branch—swapping options for the first dataset, and using a branch and bound search, keeping minimal trees only by furthest addition sequence for the second dataset. Robustness of the branches was tested by bootstrap analyses (Felsenstein 1985) for the MP (first and second datasets: 10,000 replications) and NJ methods (first and second datasets: 100,000 replications). For Bayesian analyses, the best—fit models (first dataset: GTR+I+G; second dataset: GTR+I) selected by the Akaike information criterion (Akaike 1974) were applied in MrModeltest 2.2 (Nylander 2004). Two runs with four chains of Markov Chain Monte Carlo (MCMC) iterations were performed for 1,000,000 generations, keeping one tree every 100 generations. The first 25% of generations were discarded as burn—in and the remaining trees were used to calculate a 50% majority— rule tree and to determine the posterior probabilities of the individual branches. The standard deviation for the two MCMC iteration runs was below 0.01, indicating convergence.

### Observation of oviposition and hatchability of eggs

To examine the oviposition and hatchability of *T. varians* eggs, copulated females (n = 4) were individually transferred to a 430 mL plastic cup during the photophase period. The number of eggs and egg masses was counted at the next photophase, and the females were transferred to a new cup. This was repeated until the females died. Egg hatchability was also recorded.

### Recording of developmental parameters during embryonic, larval, and pupal stages

The time (days) required for the development of each stage was recorded at three temperatures (20, 25, and 30 °C). Newly laid eggs (within one hour of oviposition) from a single female moth were divided into three groups (n = 71–83), incubated at different temperatures, and observed daily to determine the duration of embryonic stage.

To determine the duration of the larval and pupal stages, neonate larvae were randomly selected from a single batch and individually reared at different temperatures in 90 mm Petri dishes that were marked with a number for identification until adult emergence. Individuals were checked daily for larval molting, pupation, and adult emergence. Although 30 neonate larvae were used for each temperature, only about half of them reached the adult stage as a result of the high mortality rate during the first larval instar. Pupal weight and sex of these individuals were also recorded.

### Heat requirements for the development of each stage

Developmental parameters were calculated based on the time (days) required for the development of each stage at three different temperatures (20, 25, and 30 °C). The reciprocal 1/d = *V*, where *V* is velocity, was plotted for different temperatures, and linear regression parameters were calculated. The developmental zero temperature (*T*_0_) was estimated from the intersection (-*a/b*) of the regression line across the temperature at which there was no growth (*V* = 0). The number of heat units required (*K*) was estimated from as *K* = (*T* - *T*_0_) *D*, where *T* is the constant temperature and *D* is mean developmental time at that temperature.

### Chromosome preparations

In both sexes, meiotic chromosome spreads were prepared from gonads of fifth instar larvae, as described previously (Imai et al. 1977). The chromosomes were stained with 3% Giemsa in 40 mM sodium potassium phosphate buffer (pH 6.8) for 20–30 min at room temperature (Imai et al. 1977).

### Polyploid nuclei preparation

The presence of the sex heterochromatin body (SB) was investigated in cells of the sucking stomach of moths as described previously (Pan et al. 1987). The sucking stomach was dissected on a glass slide using forceps. Tissues were then stained in 3% aceto—orcein for 10 min, covered with a coverslip, and squashed. Highly polyploid nuclei in these cells were observed for the presence of SB under a light microscope at a magnification of 200–400×.

### Data deposition

Sequence data were deposited in the GenBank/EMBL/DDBJ database under the following accession numbers: *COI* of *T. varians* (Ishigaki Island), AB605613; *COI* of *E. moorei* (Wulai, Taiwan), AB605614; and *DDC* of *T. varians* (Ishigaki Island), AB605615.

## Results

### Molecular phylogeny of *T. varians*

In order to examine the phylogenetic position of *T. varians* based on the *DDC* gene, a total of 35 operation units (OTUs) were analyzed consisting of six species of Bombycinae, two species of Phiditiinae, two species of Prismostictinae, two species of Apatelodinae, eight species of Saturnidae, four species of Sphingidae, one species of Eupterotidae, three species of Anthelidae, one species of Carthaeidae, one species of Mirinidae, one species of Endromidae, one species of Brahmaeidae, and one species of Lemoniidae as the in—group taxa (Bombycoidea (*s. str*.) *sensu* Regier *et al*. (2008a)), with one species each of Mimallonidae (Mimallonoidea) and Geometridae (Geometroidea) as the out—group taxa. Phylogenetic trees yielded by NJ, MP, and Bayesian methods formed almost the same topologies. The phylogenetic tree resulting from the Bayesian analysis is shown in Figure 1. In the tree, *Trilocha* was regarded as the sister to the clade (*Bombyx* + *Ernolatia*) that was weakly supported with rather low value (posterior probabilities (PP) 0.59). On the other hand, *Trilocha* was the sister to *Ernolatia* in the trees produced by the MP and NJ methods, and the monophyly of the sister relationship was supported with relatively low values (bootstrap values (BV) 53–72%, data not shown). Bombycini (the clade comprising *Trilocha, Bombyx*, and *Ernolatia*) was strongly supported in all three analyses (BV 96–100%, PP 1.00), and Bombycinae (the clade (Bombycini + (Epiini: *Colla* + *Quentalia*) was supported with a wide range of support values (BV 51–83%, PP 1.00). Bombycinae was the sister—group to the clade consisting of Apatelodinae (*Apatelodes* + *Olceclostera*) of Bombycidae in the NJ and Bayesian methods, and the clade (Bombycinae + Apatelodinae) was the sister to the clade (Saturniidae + Eupterotidae), though the support values of the two sister relationships were low (Bombycinae + Apatelodinae: BV < 50%, PP = 0.82; (Bombycinae + Apatelodinae) + (Saturniidae + Eupterotidae): BV < 50%, PP 0.58). In all analyses, the Bombycidae *sensu* Minet (1994) emerged as polyphyletic, as previously reported (Regier et al. 2008a; Regier et al. 2008b; Zwick et al. 2011). Although the Bombycoidea (*s. str*.) *sensu* Minet (1994) was regarded as paraphyletic because of inclusion of Anthelidae (Lasiocampoidea), monophyly of the Bombycoidea (*s. str*.) *sensu* Regier et al. (2008a) was barely supported with a wide range of bootstrap values (BV 51–71%, PP 0.98).

To determine the phylogenetic position of *T. varians*, a total of 10 OTUs consisting of five species (eight individuals) of Bombycini (Bombycinae) were subsequently analyzed as the in—group, with *Apatelodes* (Apatelodinae, Bombycidae) and *Antheraea* (Saturnidae) as the out—groups (Figure 2). The phylogenetic tree produced by the Bayesian analyses of the second dataset for *COI* gene is presented in Figure 2B. The same topologies were found using NJ and MP methods. Monophyly of Bombycini (Bombycinae) was supported with low values (BV 60–62%, PP 0.67) in all analyses. This cluster first diverged into two reciprocally monophyletic groups: a clade comprising *B. huttoni, B. mori*, and *B. mandarina*, and the other consisting of *T. varians* + *E. moorei*. The monophyly of the former clade was supported with a wide range of bootstrap values (BV < 50–89%, PP 0.94), although *B. mandarina* was regarded as paraphyletic. The monophyly of the latter clade was supported with relatively high values (BV 83–85%, PP 0.88). The genetic distances between three geographic populations of *T. varians* were examined, i.e., Taipei, Ishigaki Island, and Okinawa Island (Figure 2A). Although the nucleotide sequences of *COI* genes were determined (1461 bp), no nucleotide differences were found among them.

**Table 1.**
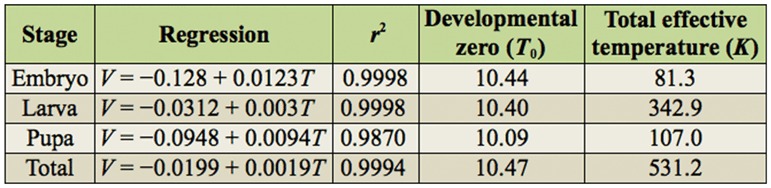
Developmental zero (°C) and total effective temperature (day–degrees) of *Trilocha varians*.

**Table 2.**
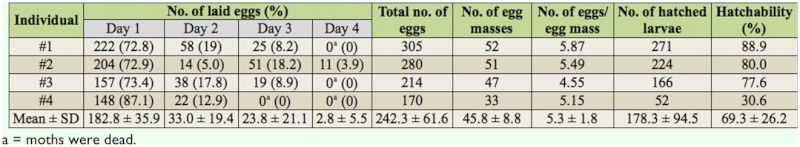
Oviposition and hatchability of *Trilocha varians* eggs.

### Laboratory rearing of *T. varians*

**Figure 3.**
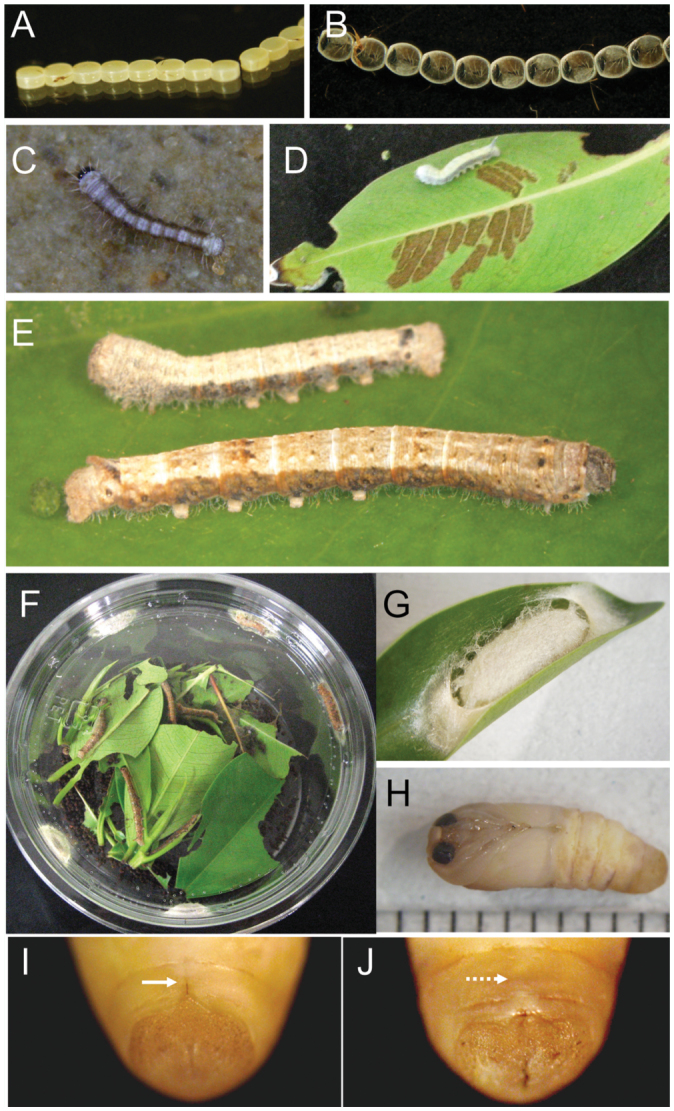
Immature stages of *Trilocha varians*. (A) Newly laid eggs of *Trilocha varians*. (B) Eggs one day before hatching. The head capsule is visible through the transparent eggshell. (C) Newly-hatched larva. (D) Third instar larva on a leaf of *Ficus microcarpa*. (E) Fifth instar larvae. (F) Last instar and spinning larvae in a 430 mL plastic cup. (G) Cocoon on a leaf of ficus *microcarpa*. (H) Male pupa. (I, J) Ventral views of abdomen of female (I) and male (J) pupae. The solid arrow indicates the suture on the ninth abdominal segment of the female, and the dashed arrow indicates the absence of the suture in the male. High quality figures are available online.

As *T. varians* adults and larvae are fairly robust, it is relatively easy to maintain laboratory stocks. Here, the morphological and behavioral characteristics of *T. varians* during the embryonic, larval, pupal, and adult stages are described (Figure 3), and tips for laboratory rearing of *T. varians* are provided. From oviposition to adult emergence, the developmental zero was 10.47 °C and total effective temperature was 531.2 day—degrees (Table 1); i.e., approximately 30 days for one generation when reared at 28 °C.

### Egg stage

Female moths reared at 25 °C laid 242.3 ± 61.6 eggs (n = 4) (Table 2). More than 70% of the eggs were laid in the first scotophase after the separation of copulating pairs. Females that laid eggs were dead by 4 days after the onset of oviposition, whereas uncopulated females lived for more than 1 week. Eggs of *T. varians* were round and cake—like in shape and laid in a line (Figure 3A), with 5.3 ± 1.8 eggs per egg mass (n = 4 moths) (Table 2). The eggs were light yellow when newly laid, turned reddish by five days after laying, and finally became black 1 day before hatching (Figure 3B). As the eggs were loosely attached, they could easily be detached from the plastic cup with the fingers. Hatching of larvae occurred in early photophase and was not usually observed in scotophase. The egg surface was disinfected by soaking the eggs in 3% formaldehyde for 5 min, a treatment that seemed to have no effect on hatchability. The developmental zero and total effective temperature during the embryonic stage was 10.44 °C and 81.3 day—degrees, respectively (Table 1).

### Larval stage

First to fourth instar larvae were white with black lines along the sides of the abdomen (Figure 3C and 3D). From the fifth instar, body color dramatically changed to reddish brown, producing an appearance similar to branches (Figure 3E). Young larvae (first to third instar) fed on the lower surface of *F. microcarpa* or *F. superba* leaves (Figure 3D), while older larvae could eat the entire thickness of the leaves, except the thick veins (see Figure 3F). Since first instar larvae were very small and active, they could wander away from a Petri dish unless it was sealed tightly. Plastic cups (430 mL) were used for rearing from the second instar to the adult stage. When the cups became crowded, the number of individuals per cup was reduced. The larvae appeared to be calm among the surrounding larvae and did not show cannibalistic or aggressive behavior. Thus, up to 30 larvae could be reared together in a single 430 mL cup during the final instar. Interestingly, the number of larval instars was affected by the rearing temperature (Table 3). When larvae were reared at a low temperature (20 °C), there was a tendency for an increase in the number of larval instars; most reached the seventh instar and became relatively larger pupae. Conversely, when larvae were reared at a higher temperature (30 °C), the number of larval instars decreased and relatively smaller pupae were produced after the fifth or sixth larval instar; no larvae entered the seventh instar. The developmental zero and total effective temperature during the larval stages was 10.40 °C and 342.9 day—degrees, respectively (Table 1).

**Table 3.**
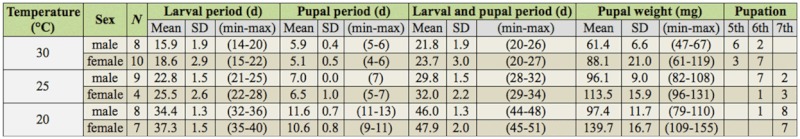
Duration of larval and pupal stages of *Trilocha varians*, and instars when they pupated.

### Cocoon and pupal stage

Cocoon color ranged between white, pale yellow, and bright yellow. Cocoons were boat—shaped and had a papery outer shell. They were typically spun on the leaves and could also be spun on the wall or cover of the rearing cup (Figure 3F and G). Spinning was completed within a day. Pupae were pale yellow with a thin, fragile exoskeleton (Figure 3H). As the backside of cocoon shells that was attached to the leaves (or plastics) was rather thin, progression of pupal and adult development could be checked from this side. Pupae turned grayish before emergence and the sex could be determined based on the presence (female) or absence (male) of a suture on the ninth abdominal segment (Figures 3I and 3J). The developmental zero and total effective temperature during the pupal stages was 10.09 °C and 107.0 day— degrees, respectively (Table 1).

### Adult (moth) stage

**Figure 4.**
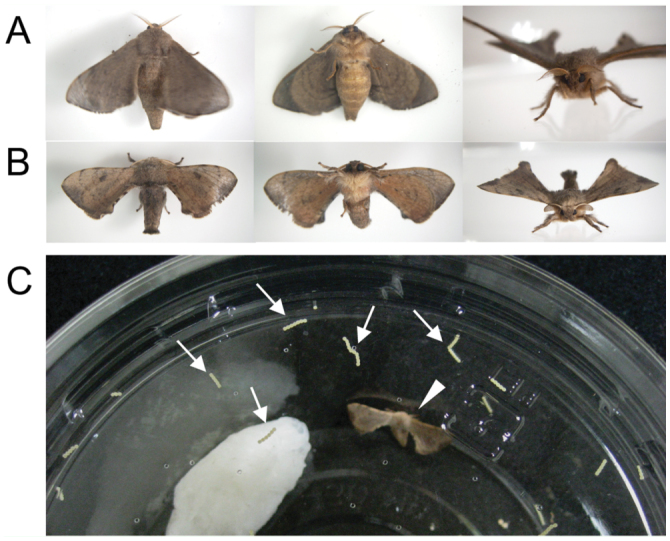
*Trilocha varians* adults. (A) Female and (B) male adults of *Trilocha varians*. (C) A female (arrowhead) laid eggs on the cover and wall of a plastic cup (arrows). High quality figures are available online.

Most adults emerged approximately 1–2 hours before the onset of scotophase, and calling behavior of females was observed during early scotophase (T. Daimon et al., unpublished). Most female moths copulated with males during the first scotophase. Copulation often lasted until late photophase (i.e., longer than 18 hours). Egg—laying behavior began at the onset of the next scotophase. When eggs were required for stock maintenance, copulating pairs were transferred to a new 430 mL cup. Moths were usually handled during photophase, when they were very inactive. As females did not refuse to lay eggs on a plastic cup (Figure 4), it was not necessary to put leaves or branches of host plants in the cup.

### Karyotype and sex chromosome of *T. varians*

**Figure 5.**
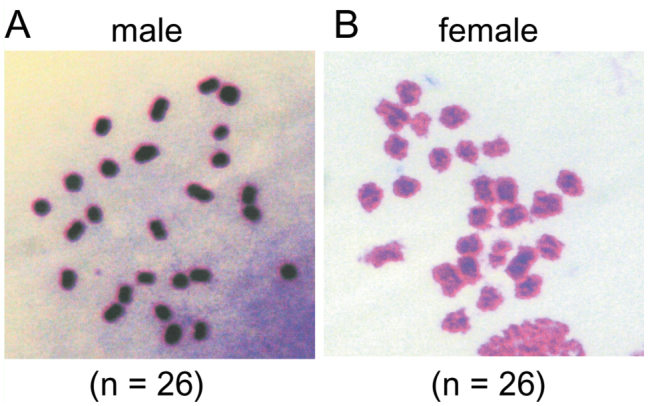
Karyotype of *Trilocha varians*. Chromosome preparations of (A) male and (B) female *Trilocha varians* larvae, showing 26 bivalent chromosomes in both sexes. High quality figures are available online.

**Figure 6.**
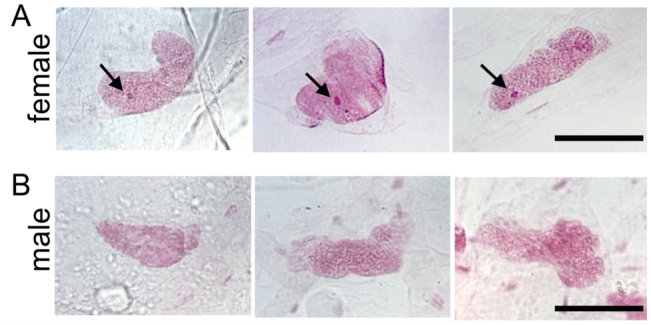
Sex heterochromatin body of *Trilocha varians* females. (A) Nuclei prepared from the sucking stomach of adult females showing a deeply strained SB (arrows). (B) Male nuclei lacking SB. Preparations from three individuals of each sex are shown. Scale bar indicates 100 µm. High quality figures are available online.

The karyotype of *T. varians* was investigated using chromosome preparations from larval gonads (Figure 5). In meiotic metaphase, 26 bivalents were observed both in males and females, demonstrating that the haploid karyotype of *T. varians* consists of n = 26 chromosomes. Sucking stomachs were prepared and inspected for the presence or absence of SB, which has been deduced to be composed of condensed W chromosomes (Ennis 1976; Traut and Marec 1996). In females, each nucleus displayed a single, spherical heterochromatin body (Figure 6A). In contrast, no heterochromatin was observed in males (Figure 6B). The presence of SB in females and its absence in males suggests a WZ/ZZ (female/male) sex chromosome system in *T. varians*.

## Discussion

The present study investigated the molecular phylogeny, development time, and karyotype of *T. varians*. Although the subfamily Bombycinae is a relatively large group and comprises approximately 20 genera (Regier et al. 2008a), knowledge of the biology of this group is very limited, except for *B. mori*, a lepidopteran model insect.

Our molecular study suggested that *Trilocha* is assigned to the Bombycini of Bombycinae (Figures 1 and 2B), as has been supposed by previous studies (Lemaire and Minet 1998; Regier et al. 2008a; Zolotuhin and Witt 2009). Phylogenetic trees based on *COI* and *DDC* genes both suggest *Trilocha* is closely related to *Ernolatia* and *Bombyx*. On the other hand, the *COI* sequences of *T. varians* detected no nucleotide differences between Taiwan and the Ryukyu Island (Figure 2B). This very low genetic variation indicates recent and/or ongoing gene flow among the three populations over geographical gaps (ocean) of about 280 km between Taiwan and Ishigaki Island, and about 400 km between Ishigaki and Okinawa Islands (Figure 2A).

The developmental zero and total effective temperature for *T. varians* were determined in this study (Table 1). As *T. varians* seems to be a nondiapausing species, there may be nine or 10 generations each year in Taipei. Although *T. varians* is a common species in Taiwan (Y.-F. Hsu, personal observation), its distribution in Japan was not reported until recently. The first record was by Kishida (2002), who collected *T. varians* male moths on Ishigaki Island in December 2001. Since then, reports of the capture of *T. varians* have increased on Ishigaki, Iriomote, and Okinawa Islands (Kishida 2011). These observations, together with our finding that *COI* sequences were 100% identical among the Taipei, Ishigaki, and Okinawa populations (Figure 2B), suggest that the distribution of *T. varians* on Ishigaki and Okinawa Islands is a consequence of a recent and/or ongoing immigration from Taiwan, probably due to global warming, typhoons, or transplanting, as is often the case with several butterfly species, such as *Pieris canidia, Chilades pandava, Catochrysops panormus, Hypolimnas anomala*, and several danaine species (Yata et al. 2007; Wu et al. 2010) . As *T. varians* larvae and moths were found in spring and winter on Ishigaki Island (Kishida 2002) and Okinawa Island (see the Materials and Methods), *T. varians* may have successfully colonized these islands.

We developed a method for laboratory rearing of *T. varians*. This species has the following advantages that allow easy maintenance of laboratory stocks: (1) it can be reared under a wide range of temperatures; (2) cannibalism or aggression between larvae was not observed; (3) no special care or apparatus is required (e.g., soil for pupation, food for moths, a large space for mating, or host plants for oviposition); (4) the entire lifecycle can be completed in a conventional Petri dish and plastic cup; and (5) little space is necessary for stock maintenance. However, there are two disadvantages: (1) host plants of *T. varians* (*Ficus* spp.) are basically subtropical and defoliate in the cold season; and (2) *T. varians* seems to not undergo diapause. As *T. varians* did not grow well on commercially available artificial diets for *B. mori* or other insects (T. Daimon and C. Hirayama, unpublished data), we are now developing an artificial diet optimized for *T. varians*.

One interesting feature of *T. varians* is that the number of larval molts was greatly affected by rearing temperature (Table 3). This variability in the number of larval instars resulted in great variation in body size, with more than a twofold difference in pupal weight in some individuals (Table 3). Such phenomena are only rarely observed in *B. mori*, in which the number of larval molts seems to be genetically fixed and is not readily affected by environmental conditions (Fukuda 1944; Kamimura and Kiuchi 2002). It is therefore of interest to investigate additional genera and species in Bombycinae.

**Table 4.**
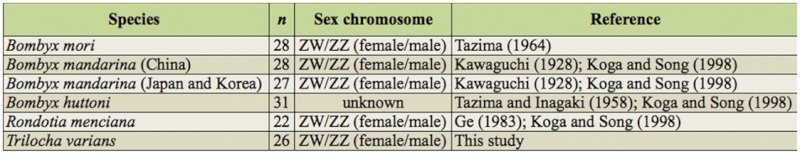
Karyotype and sex chromosome of the tribe Bombycini.

To our knowledge, all species of the tribe Bombycini feed on the family Moraceae in nature (Zolotuhin and Witt 2009). *Trilocha varians* larvae feed on the genus *Ficus*, while *B. mori* feed on the genus *Morus* (mulberry trees). Mulberry leaves are toxic to insects that do not feed on them (Konno et al. 2006). This toxicity is mainly conferred by its sugar— mimic alkaloids, such as 1, 4-dideoxy-1, 4-imino-D-arabinitol (D-AB1) and 1-deoxynojirimycin (DNJ), which occur in mulberry latex at extremely high concentrations (Konno et al. 2006). Recent studies have demonstrated that *B. mori* has developed a unique enzymatic adaptation to circumvent the toxic effects of mulberry alkaloids (Hirayama et al. 2007; Daimon et al. 2008). Therefore, we questioned whether *T. varians* could also feed and grow well on mulberry leaves. Although newly—molted second instar *T. varians* larvae ate a small amount of mulberry leaves on the first and second days, they did not grow well and died within 5 days (T. Daimon, unpublished data). This suggests that *T. varians* does not have mechanisms that provide tolerance to the defense chemicals of the mulberry. Thus, it would be interesting to examine whether the latex of *F. microcarpa* contains sugar—mimic alkaloids. As most species of the subfamily Bombycinae feed on the genus *Ficus* (Zolotuhin and Witt 2009), the ability for feeding on *Morus* might be a novel trait among the Bombycinae.

Most lepidopteran species are reported to have a haploid number close to 30 (Robinson 1971). Thus, the chromosome number of *T. varians* (n = 26, Figure 5) seems close to the average. Four species of the tribe Bombycini have been karyotyped to date (Table 4) (Kawaguchi 1928; Tazima and Inagaki 1958; Tazima 1964; Ge 1983; Koga and Song 1998). Their chromosome numbers vary from n = 22–31, indicating the chromosome fusion and fission after divergence from the tribe Epiini, which is distributed in Central and South America. As the chromosome number of *T. varians* is close to that of *B. mori*, there seems to be a high degree of synteny conservation between *T. varians* and *B. mori*. It is also noteworthy that the chromosome number of *B. mandarina* varies among geographic populations. It is thus of interest to examine whether such a geographic variation is also present in *T. varians*. The WZ/ZZ sex chromosome system is the most common type among Lepidoptera, while the Z/ZZ is less frequent. The Z/ZZ system is generally found in basal linages, but is also found in some species belonging to more advanced groups, e.g., the Eri silkworm, *Samia cynthia ricini* (Satuniidae) (Yoshido et al. 2005). Thus, we investigated the sex chromosome of *T. varians* and found that this species has a WZ/ZZ sex chromosome. Despite the difference in chromosome number, a WZ/ZZ (female/male) sex chromosome system seems common in Bombycini.

In conclusion, this study described the biology of *T. varians*. Comparative studies on ecological traits (e.g., host plant selection, tolerance to mulberry's toxic latex, sex pheromone systems, and diapause systems) between *T. varians* and *B. mori* will contribute to discovery of the molecular mechanisms underlying the ecology and evolution of insects. Recent advances in next—generation sequencing (NGS) technologies (Metzker 2010) will facilitate comparative genomics analyses among insect species. We are currently conducting large—scale comparative transcriptome analyses between *T. varians* and *B. mori* using the NGS technology to shed light on the ecological traits of the bombycid moths and to elucidate their evolutionary history.
